# Deficits in sensory prediction are related to delusional ideation in healthy individuals

**DOI:** 10.1016/j.neuropsychologia.2010.10.024

**Published:** 2010-12

**Authors:** Christoph Teufel, Arjun Kingdon, James N. Ingram, Daniel M. Wolpert, Paul C. Fletcher

**Affiliations:** a Brain Mapping Unit, Behavioural and Clinical Neuroscience Institute, Department of Psychiatry, University of Cambridge, UK; b Computational and Biological Learning, Department of Engineering, University of Cambridge, UK

**Keywords:** Motor control, Delusions, Sensory prediction, Schizophrenia, Schizotypy

## Abstract

Motor control strongly relies on neural processes that predict the sensory consequences of self-generated actions. Previous research has demonstrated deficits in such sensory-predictive processes in schizophrenic patients and these low-level deficits are thought to contribute to the emergence of delusions of control. Here, we examined the extent to which individual differences in sensory prediction are associated with a tendency towards delusional ideation in healthy participants. We used a force-matching task to quantify sensory-predictive processes, and administered questionnaires to assess schizotypy and delusion-like thinking. Individuals with higher levels of delusional ideation showed more accurate force matching suggesting that such thinking is associated with a reduced tendency to predict and attenuate the sensory consequences of self-generated actions. These results suggest that deficits in sensory prediction in schizophrenia are not simply consequences of the deluded state and are not related to neuroleptic medication. Rather they appear to be stable, trait-like characteristics of an individual, a finding that has important implications for our understanding of the neurocognitive basis of delusions.

## Research highlights

▶ Levels of sensory-motor predictions vary gradually in the healthy population. ▶ Deficient predictions are related to a delusion-like style of thinking. ▶ The continuum between health and psychosis exists even on a sensory-motor level.

## 1 Introduction

Predicting the sensory consequences of self-generated action is a key component of motor control (Wolpert & Flanagan, 2001). Of particular importance in the current context is the notion that the sensory predictions that are thought to arise from an efference copy of the motor command form a basis for action attribution. Specifically, if the predicted sensation of a movement corresponds to the actual sensory feedback – as is the case with self-generated actions – we experience agency. By contrast, a mismatch between prediction and sensation suggests an external source. An additional aspect of this prediction process is that the predicted sensory consequences of an action are subtracted from the actual feedback, partially canceling out sensory changes that are due to self-generated movement (Bays, Flanagan, & Wolpert, 2006; Bays and Wolpert, 2007; Bays, Wolpert, & Flanagan, 2005; Blakemore, Wolpert, & Frith, 1998; Lindner, Haarmeier, Erb, Grodd, & Thier, 2006; Shergill, Bays, Frith, & Wolpert, 2003). This cancellation attenuates predicted sensation, thereby accentuating more relevant unpredicted sensory information. Predictive attenuation explains why the tactile stimulation that we perceive when we touch ourselves is weaker than the same stimulus externally imposed (Bays and Wolpert, 2007; Bays et al., 2005, 2006; Shergill et al., 2003) or why we cannot tickle ourselves (Blakemore et al., 1998).

Deficits in sensory-predictive processes have been linked to specific symptoms in psychopathology, most notably delusions of control in schizophrenic patients (Frith, Blakemore, & Wolpert, 2000; Lindner, Thier, Kircher, Haarmeier, & Leube, 2005; Shergill, Samson, Bays, Frith, & Wolpert, 2005; Synofzik, Thier, Leube, Schlotterbeck, & Lindner, 2010). Patients suffering from such delusions experience their own actions as being made for them by an external agent rather than by their own will. Intriguingly, however, the actions that are experienced as forced upon them are in line with the patient's intentions. The deficits underlying delusions of control are thus not related to an inability to initiate an intended action but to register this action as internally triggered. Specifically, it is thought that a failure to predict the sensory consequences of one's own actions leads to a mismatch or prediction-error so that actual sensory feedback is surprising, resulting in the feeling that the action was not internally generated. Persistence of prediction-error would lead to a reduced sense of agency, which is especially noteworthy given that many of the first-rank symptoms of schizophrenia seem to reflect external attributions of internally generated phenomena (Fletcher & Frith, 2009).

Although there is evidence that schizophrenic patients show deficits in predicting the sensory consequences of self-generated actions (Lindner et al., 2005; Shergill et al., 2005; Synofzik et al., 2010), such case–control studies can be problematic due to medication effects. A complementary approach to exploring such symptom models involves assessing schizotypal characteristics in healthy people, treating schizophrenia as an extreme expression of a continuous phenotype normally distributed in the population (Chapman, Chapman, Kwapil, Eckblad, & Zinser, 1994; Claridge, 1994; Crow, 1998; Meehl, 1962; Peters, Joseph, Day, & Garety, 2004; Peters, Joseph, & Garety, 1999). Besides being of inherent importance for the conceptualization of schizophrenia, such an individual-differences approach offers new ways of testing models of symptoms in the absence of medication. The approach may also provide an opportunity to distinguish between state-like and trait-like contributions to symptoms.

In this study, we explored the sensory-prediction model of delusions using an established force-matching task (Bays & Wolpert, 2007; Bays et al., 2005, 2006; Shergill et al., 2003), designed to quantify the degree of sensory attenuation arising from predictive processes. We related variability in predictive attenuation to delusion-proneness and schizotypy in healthy participants. Given the deficits in sensory prediction seen in schizophrenic patients (Lindner et al., 2005; Shergill et al., 2005; Synofzik et al., 2010), we predicted a negative correlation between levels of sensory prediction and the tendency towards delusional ideation.

## 2 Methods

### 2.1 Participants

Thirty healthy participants (18 women; age range 18–25 years) gave written, informed consent. Two subjects were excluded following testing due to performance on the force-matching task deviating from the group mean more than 2 standard deviations. The remaining participants were manly right-handed; the mean score on the Edinburgh Handedness Inventory (Oldfield, 1971) was 73.7 (*S.E.* 6.5).

### 2.2 Force-matching procedure

**Fig. 1:**
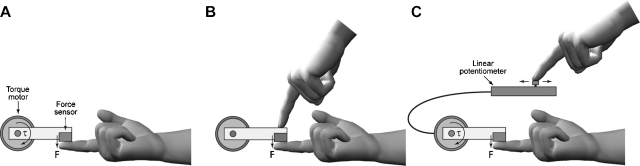
The force-matching task. (A) The force actuator consisted of a lever attached to a torque motor which was under computer control. A sensor at the end of the lever measured the force applied to the subject's finger. In the first part of each trial, the actuator generated a target force on the subject's left index finger. In the second part of each trial, the subject was required to match the target force. (B) In the Finger condition, the subject was required to directly match the target force by actively pressing on the top of the lever with their right index finger. In this condition, the brain can predict the sensory consequences of the finger movement based on an efference copy of the motor command and attenuate the predicted sensation. Depending on the extent of this predictive attenuation, a larger active force is therefore required in order to match the percept of the previously experienced target force. (C) In the Slider condition, the subject was required to indirectly match the target force by moving a linear potentiometer which controlled the force generated by the actuator. Here, no sensory prediction is possible due to the unusual relationship between action and sensory consequences.

In the force-matching task (Shergill et al., 2003) a force was applied to the participants’ right index finger by a lever that was attached to a torque motor (Fig. 1). Subjects subsequently had to match this passively experienced target force by one of two means: Either they used their left index finger to directly exert force onto the lever, pressing onto their right index finger (“Finger condition”); or else they controlled the lever indirectly by sliding a linear potentiometer that controlled the torque motor (“Slider condition”; the gain of the slider was 0.5 N/cm). In the Finger condition, central processes in the brain can use an efference copy of the motor command in order to predict and attenuate the sensory consequences of the movement. The attenuation of the predicted sensation can be measured as overcompensation when participants try to match the sensation of a previous force. In other words, because sensation in the Finger condition can be predicted, and is therefore attenuated, participants should consistently apply a larger force onto their finger when trying to match the percept of the passively experienced target force (Bays & Wolpert, 2007; Bays et al., 2005, 2006; Shergill et al., 2003). The Slider condition serves as a control due to the unusual relationship between the action (moving the slider in the horizontal plane) and its sensory consequences (feeling a force applied to one finger of the other hand). Because participants have no prior experience with the slider, and exposure during the experiment is limited to 80 trials, there should be no or almost no predictive processes to attenuate perception. Participants should thus be able to match the force more accurately than in the Finger condition.

All subjects completed 80 trials in each condition. Ten different target forces (8 trials of each), increasing in increments of 0.25 N from 0.50 N to 2.75 N, were randomly presented. The order of conditions was counterbalanced across participants.

### 2.3 Questionnaires

Participants completed the 21-item Peters et al. Delusion Inventory (PDI) (Peters et al., 2004) and the Magical Ideation Scale (MgI) (Eckblad & Chapman, 1983). Both of these inventories are schizotypy scales specifically designed to quantify delusion-like ideas in the general population. One-tailed tests were used in view of the clear predictions relating sensory prediction and delusional ideation.

In a more exploratory analysis, we investigated perceptual aberrations and the sense of internal versus external control related to events in everyday life. Each participant completed the 35-item Perceptual Aberration Scale (PAS) (Chapman, Chapman, & Raulin, 1978) and a Locus of Control questionnaire (LC) (Levenson, 1973). The latter quantifies the extent to which participants localize control over important events in everyday life internally (“Internal”) or attribute it externally either to chance (“External Chance”) or to a powerful other person (“External Other”). The influence of handedness on performance in the force-matching task is unknown. Nevertheless, in order to control for any potential effects, the Edinburgh Handedness Inventory (EHI) (Oldfield, 1971) was also completed.

Normality of all behavioral and psychometric data was explored by inspection of normal Q–Q plots and by using the Kolmogorov–Smirnov test with Lilliefors corrections. To allow uniform analyses, skewed data were subjected either to a square root transformation if they were positively skewed (MgI and PAS) or a reversed square root transformation if they were negatively skewed (EHI).

## 3 Results

### 3.1 Sensory prediction

**Fig. 2:**
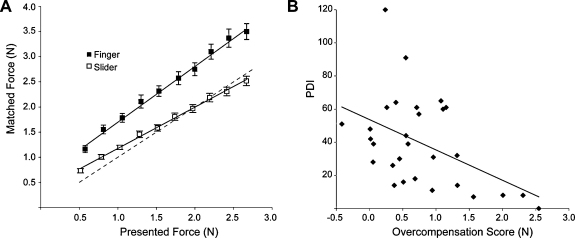
(A) Mean force (±*S.E.*) applied by the participants as a function of the mean presented target force. Results of the Finger condition are represented by dark squares and those of the Slider condition by white squares. The dotted line indicates perfect performance. The amount of overcompensation (i.e., the application of a larger force than the previously experienced target force) is a direct measure of sensory-predictive processes. (B) The participants’ tendency towards delusional ideation (as measured by the PDI) as a function of their sensory prediction (as measured by their mean overcompensation score).

Consistent with previous results (Shergill et al., 2003), when trying to match a passively experienced target force, participants applied a larger force in the Finger condition than the Slider condition (Fig. 2A). A comparison of the regression lines of the force-matching performance of each participant indicated that both the intercept (paired-sample *t*-test, *t* = −3.70, *df* = 27, *p* ≤ .001) and the slope (*t* = −4.14, *df* = 27, *p* ≤ .001) were significantly larger in the Finger than the Slider condition. Across participants, the intercept increased on average by 0.33 N (*S.E.* 0.08 N) and the slope by 41% (*S.E.* 12%).

### 3.2 Relation between sensory prediction and delusional ideation

Given that the PDI and the MgI are both measures of delusional ideation, it is unsurprising that a partial correlation analysis controlled for handedness showed that their scores were significantly related to each other (*r* = .46, *p* ≤ .01), a finding consistent with previous studies (Peters et al., 1999). In order to relate the questionnaires to sensory prediction, we calculated a composite score for each participant in the force-matching task by subtracting the average force applied in the Slider condition from that applied in the Finger condition. This overcompensation measure quantifies the degree of sensory prediction. A partial correlation analysis controlled for handedness showed no relation between the participants’ transformed MgI scores and their overcompensation scores (*r* = −.25, *n.s.*, one-tailed). PDI scores, however, were significantly negatively correlated with overcompensation scores (*r* = −.42, *p* = .015, one-tailed) (Fig. 2B). A Kendall's partial correlation controlled for handedness, which is insensitive to outliers, confirmed this finding (*Kendall's Tau* = −0.25, *p* < 0.05, one-tailed). *p*-Values were estimated based on 1000 permutations.

Given that the PDI and the MgI are non-independent, Bonferroni correction would be inappropriate and too conservative. Note, however, that the significant relation between overcompensation and PDI would survive even such correction and would consequently survive any correction that would take the non-independence into account.

### 3.3 Perceptual aberration and locus of control

There was no relationship between aberrant perceptual experiences (as measured by the transformed PAS scores) and overcompensation scores (*r* = .01, *n.s.*). Analysis of the LC questionnaire indicated no relation with any of the subscales (Internal: *r* = .05, *n.s.*; External Chance: *r* = .07, *n.s.*; External Other: *r* = −.35, *p* = 0.077). Again, the analyses are partial correlations controlled for handedness.

## 4 Discussion

Our study demonstrates a significant relationship between reduced prediction of the sensory consequences of self-generated movement and a tendency towards delusional ideation in healthy participants: the more accurate participants were in matching an experienced force (i.e. the lower their predictive attenuation), the higher they scored on the PDI. This finding is consistent with the notion that a continuum between health and psychosis extends even to a basic sensory level.

It is perhaps unsurprising that sensory prediction correlated with delusional ideation as measured by the PDI but not by the MgI, given that the latter has been criticized for under- as well as over-estimating delusional ideation in the general population (Peters et al., 2004). The PDI was constructed to resolve the problems of the MgI and to provide a more accurate measure of delusional ideation in healthy participants (Peters et al., 1999, 2004). None of our exploratory analyses regarding perceptual aberration or the patients’ localization of control showed a significant result, highlighting the specificity of the relation between deficits in sensory prediction and delusional ideation.

Although only longitudinal data can provide definitive evidence, our results suggest that deficits in sensory prediction represent a stable, trait-like characteristic of individuals, varying continuously in the general population. Given that the abnormal sensory experiences associated with such deficits are thought to contribute critically to the emergence of delusions of control in patients (Fletcher and Frith, 2009; Frith et al., 2000; Lindner et al., 2005; Shergill et al., 2005; Synofzik et al., 2010), this characteristic may predispose to certain styles of reasoning and types of experience in healthy people, which in turn may ultimately predispose to the emergence of delusions under certain circumstances. Furthermore, our results suggest that the deficient sensory-predictive processes in psychosis shown by previous studies (Lindner et al., 2005; Shergill et al., 2005; Synofzik et al., 2010) are unlikely to be a confound of neuroleptic medication.

As with previous studies, participants were able to match an experienced force accurately when they used the slider to control the lever that pushed onto their passive finger, indicating that they did not predict and attenuate the sensory consequences of their action in this condition. An interesting question for future research is whether extensive experience with the slider would allow participants to incorporate the predictive relation between the position of the slider and the experienced force into their sensory predictions. Over time, this should lead to increasing sensory attenuation and consequently to overcompensation even in the slider condition. Alternatively, an additional requirement for sensory attenuation might be that the relation between an action and its sensory consequences conforms to a physically plausible causal scenario, in which case no change in sensory prediction and attenuation should be seen in the slider condition even after extensive training.

To conclude, the finding that variability in sensory prediction characterizes the general population, and is related to delusional ideation, supports the sensory-prediction model of delusions and complements work with psychotic patients (Lindner et al., 2005; Shergill et al., 2005; Synofzik et al., 2010). More generally, this observation highlights the usefulness of an individual-differences approach to assess specific symptom models of schizophrenia. This opens up exciting possibilities for future work to characterize the neural signature of fundamental processes that contribute to this mental illness using imaging techniques and to track their neurodevelopmental trajectory.
